# Supplementary material for: Spontaneous Mutations in Saccharomyces cerevisiae mtDNA Increase Cell-to-Cell Variation in mtDNA Amount
Source: Int J Mol Sci. 2023 Dec 12;24(24):17413. doi: 10.3390/ijms242417413 (PMC10743915; doi:10.3390/ijms242417413)
Supplement: Supplementary file 1 [file ijms-24-17413-s001.zip › ijms-2695885-supplementary.pdf]

Supplementary materials: Spontaneous mutations in *Saccharomyces cerevisiae* mtDNA increase cell-to-cell variation in mtDNA amount

Elena Yu. Potapenko[1], Nataliia D. Kashko[1], Dmitry A. Knorre[2]

[1] Faculty of Bioengineering and Bioinformatics, Lomonosov Moscow State University, Russia
[2] Belozersky Institute of Physico-Chemical Biology, Lomonosov Moscow State University, Russia

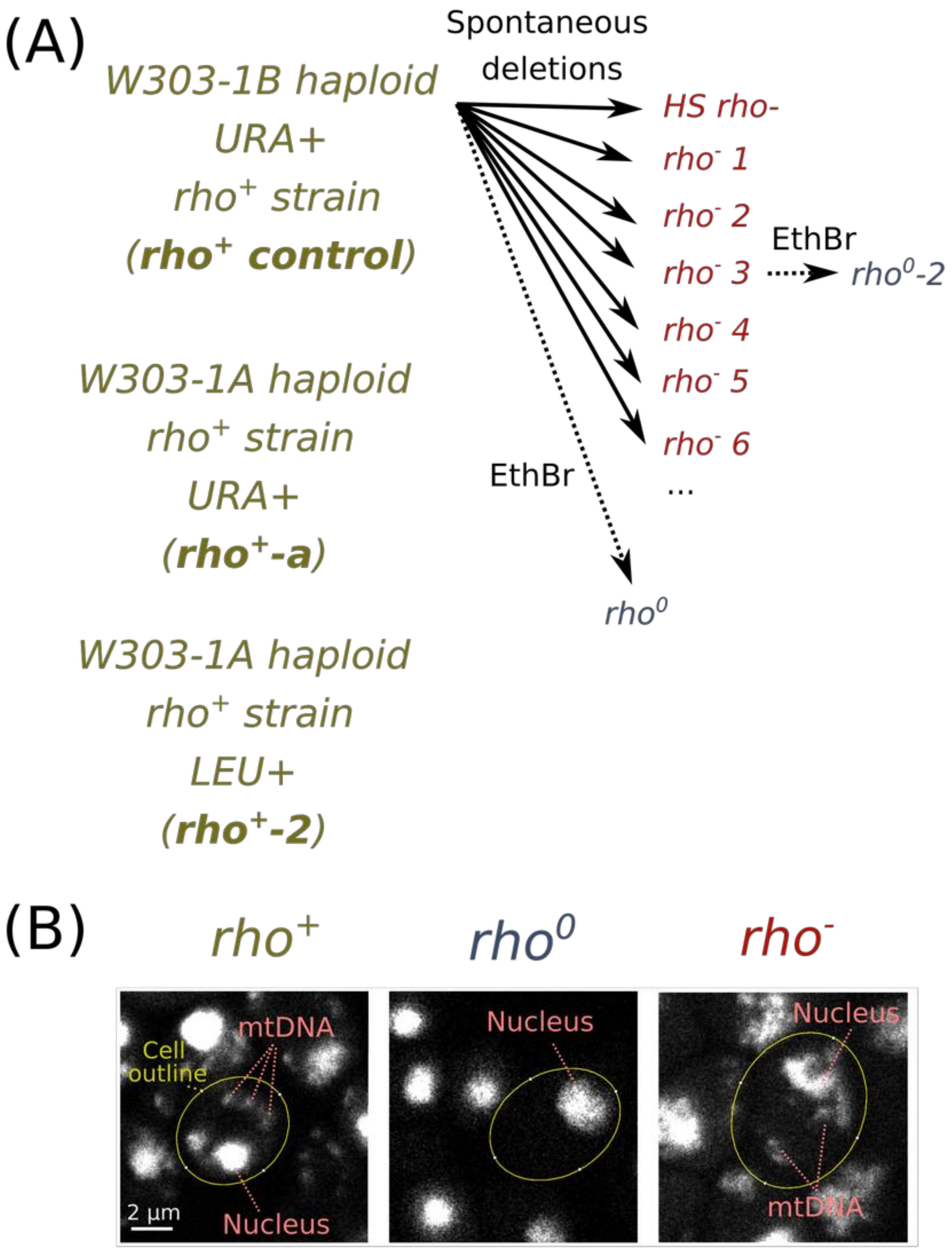

Figure S1. (A) Strains used in the study. Dotted line (EthBr) indicates depletion of mtDNA with ethidium bromide (see Materials and methods). *HS* (hypersuppressive) *rho*$^-$ strain was obtained previously (Karavaeva et al. 2017), while the rest of the *rho*$^-$ strains were selected at random in this study. (B) DAPI staining of *rho*$^+$, and *rho*$^-$ cells, a representative photograph shows that cytoplasm of *rho*^{0} cells is clear from DAPI foci.

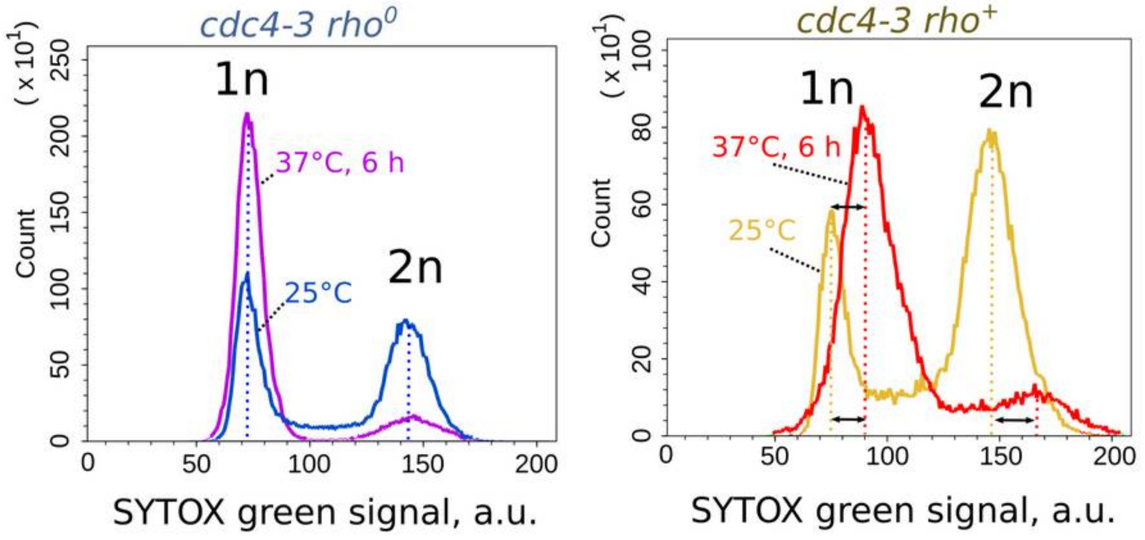

Figure S2. A representative flow cytometry diagram showing SYTOX green intensity signal of *cdc4-3 rho*^{0} (left) and *cdc4-3 rho*$^+$ (right). Yeast cells were cultivated until they reached exponential growth phase in YPD, 25°C. Subsequently, they were incubated for a 6-hour period at either permissive (25°C) or non-permissive (37°C) temperature.

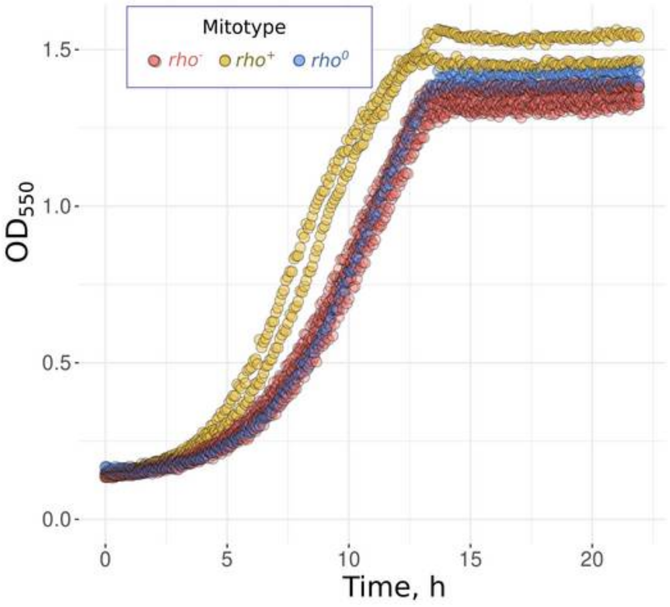

Figure S3. Growth kinetics of *rho*$^-$, *rho*^{0} and *rho*$^+$ cells in liquid YPD, 30°C. Optical density was measured each five minutes in SPECTROstar Nano microplate reader.

(A)

$$rSD = \left(\text{Median of}\left\{\left|X_i - Median_x\right|\right\}\right) \times 1.4826$$

$$\text{rCV} = \frac{rSD}{Median_x} \qquad \%\,\text{rCV} = \frac{rSD}{Median_x} \times 100\%$$

(B)

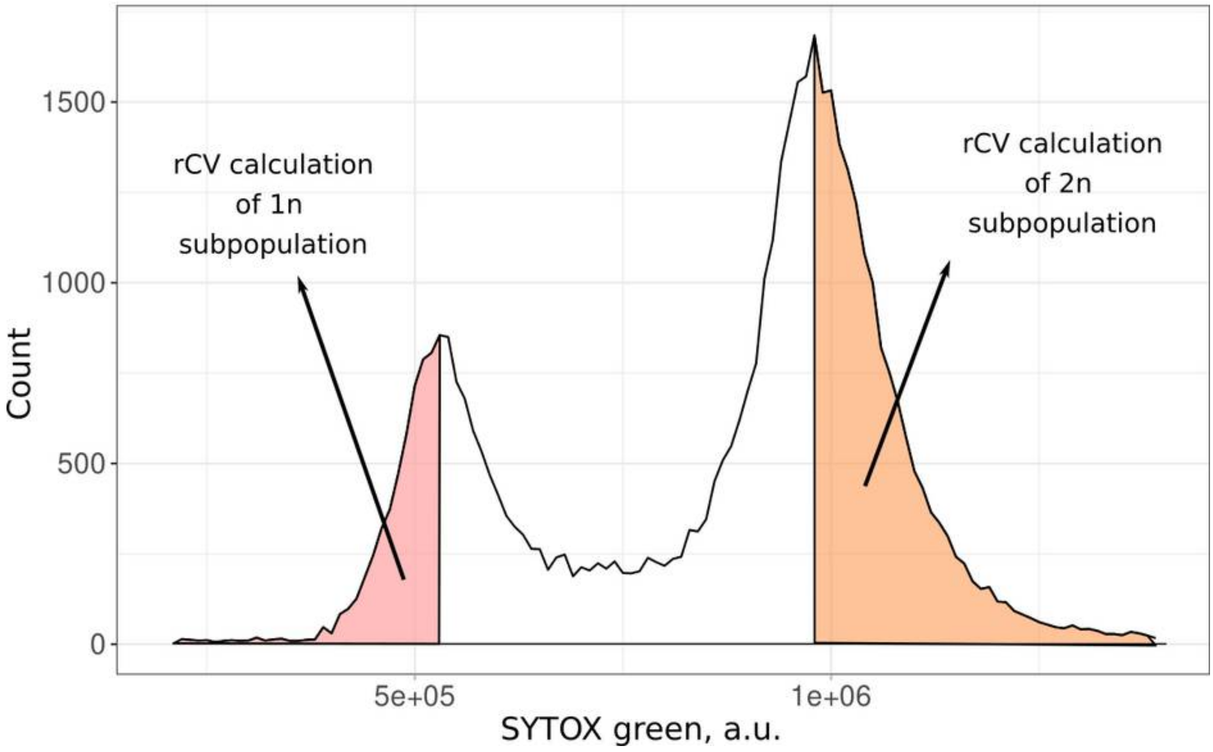

Figure S4. Equations used to calculate robust coefficient of variation (A) and illustration showing subpopulations selected for analysis of rCV in 1n and 2n subpopulations (B).

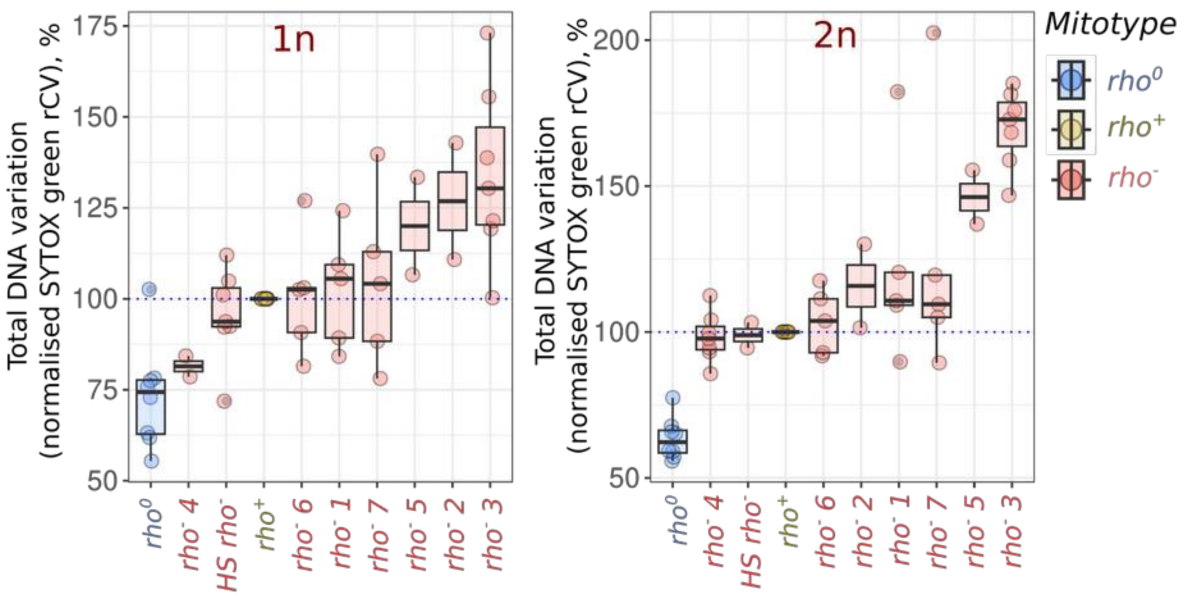

Figure S5. *Rho*⁻ strains show increased rCV of total DNA content in 1n (G1 phase) and 2n (G2/M) exponentially grown yeast cells at 25°C.

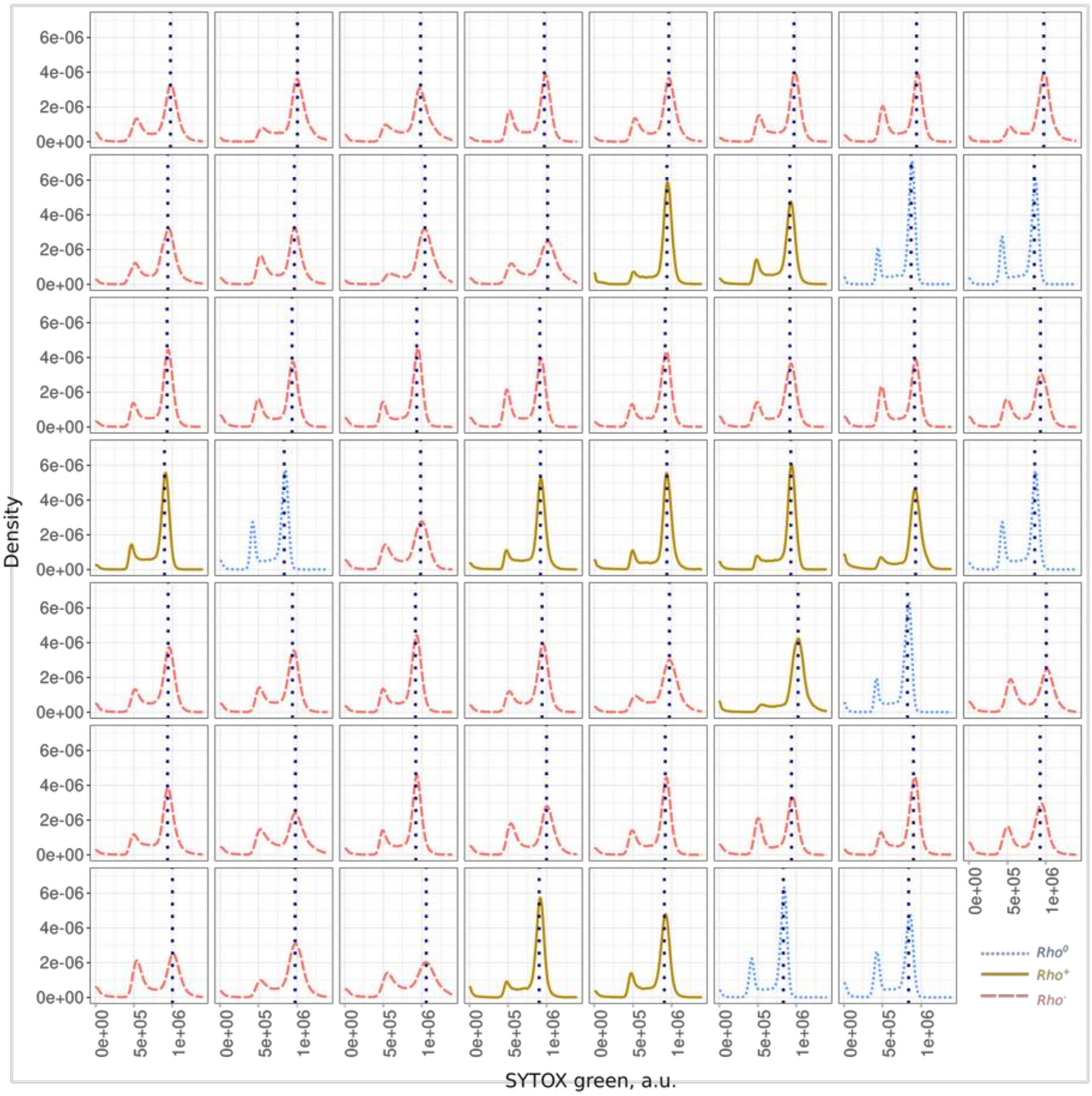

Figure S6. Positions of 2n peaks identified using multimode R package. Each panel represents a separate experiment; the vertical dotted line represents the position of the identified 2n peak position. We took events with SYTOX green signal above this threshold to study the interrelation of cell size (FSC-A) and SYTOX green signal cell-to-cell heterogeneity.

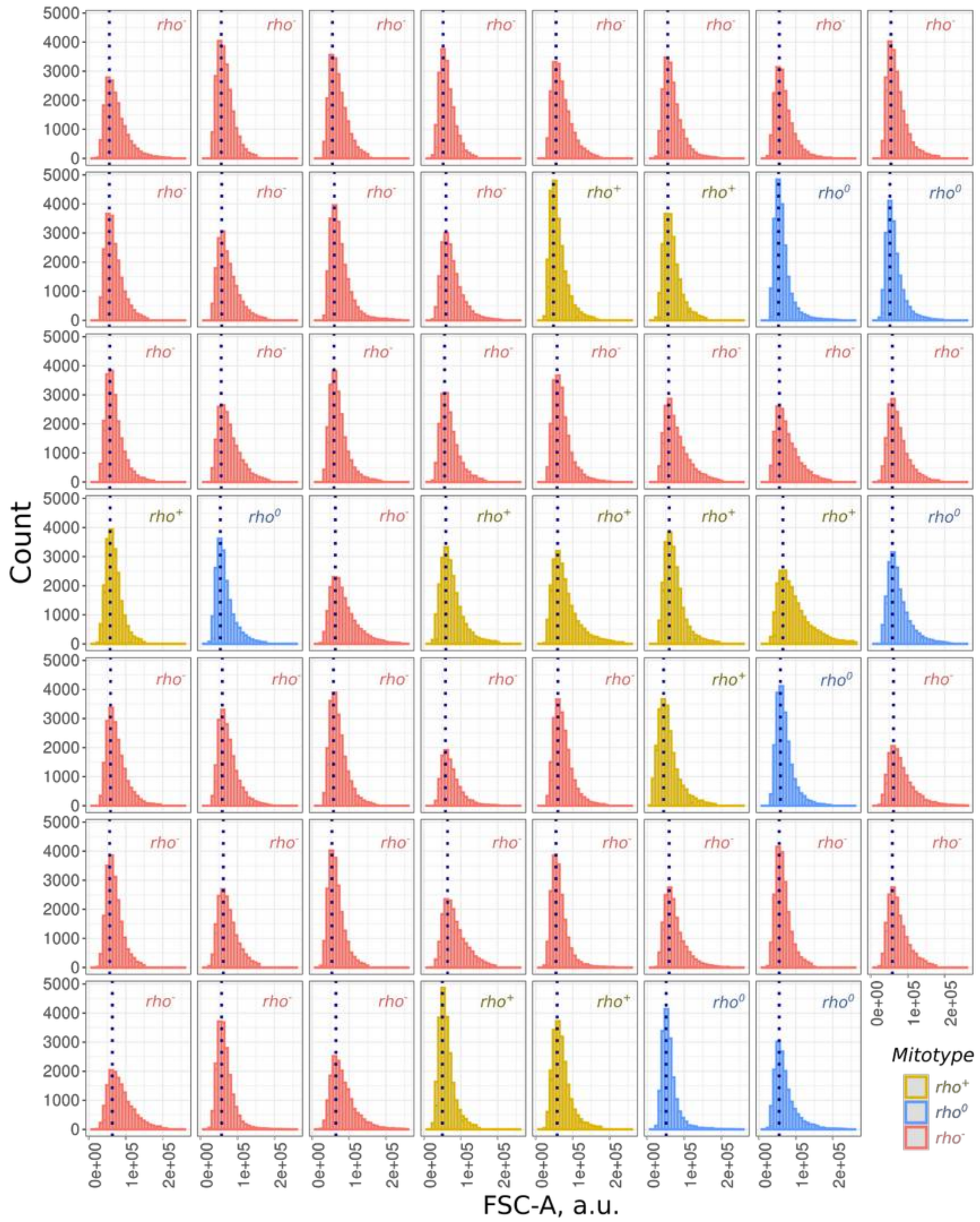

Figure S7. Distributions of cell sizes of 2n yeast cells with the SYTOX signal above the threshold in figure S6. Dotted line shows the position of the distribution modes.

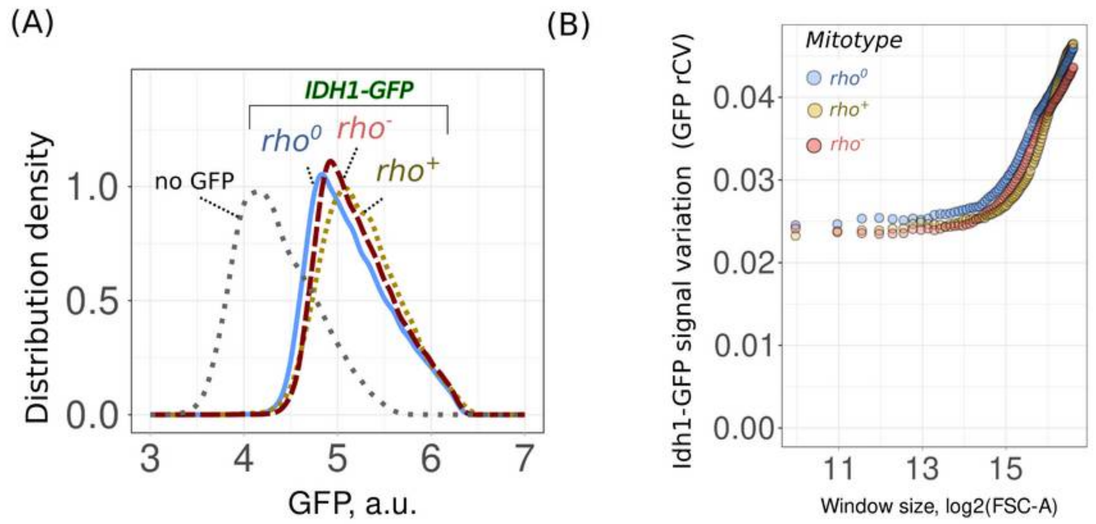

Figure S8. Variation in Idh1-GFP levels across *rho*^{0}, *rho*$^{-}$ and *rho*$^{+}$ cells. (A) Representative result of a flow-cytometry experiment with live cells. "no GFP" histogram represents autofluorescence of wild-type *rho*$^{+}$ cells without Idh1-GFP; (B) Analysis of the intrinsic variance of Idh1-GFP, comparable to the analysis presented in Figure 4 of the manuscript.

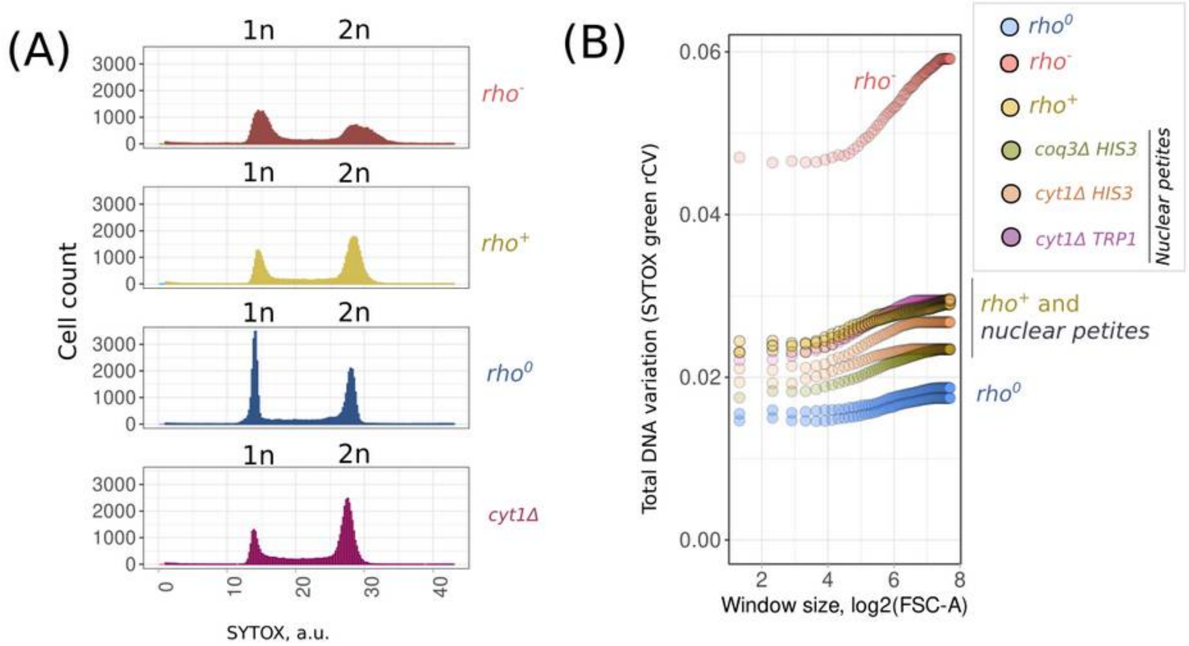

Figure S9. SYTOX signal and rCV of nuclear petites. (A) SYTOX green intensity signal of *rho*^{0} , *rho*$^{-}$, *rho*$^{+}$ and nuclear petite *Δcyt1 HIS3* cells. (B) rCV values across different cell size ranges of the wild type *rho*$^{+}$, *rho*$^{-}$*3*, *rho0* and nuclear petite strains*: coq2Δ, cyt1Δ HIS3* and *cyt1Δ TRP1*. A.u. refers to arbitrary units. For this experiment, yeast cells were cultivated up to exponential growth phase in YPD, 30°C.

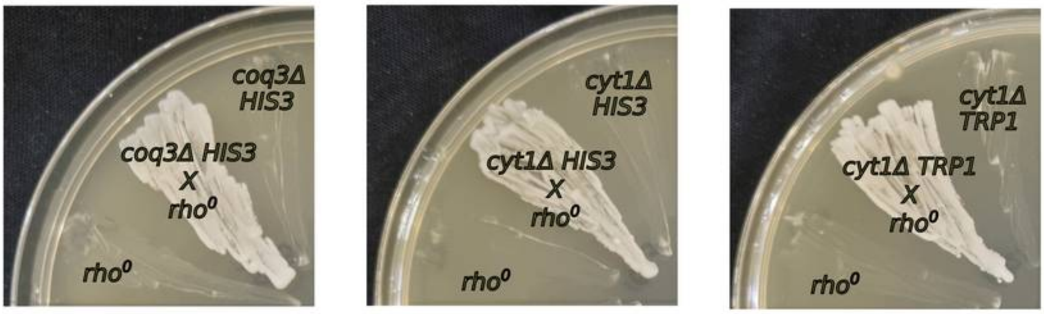

Figure S10. Nuclear petites *W303-1A cyt1Δ::HIS3, cyt1Δ::TRP1, coq3Δ::HIS3* retain mtDNA. The photograph shows the sectors of YPGly Petri dishes with streaks of *rho*^{0} and nuclear haploid strains, along with the diploid strains generated by their crossings (24 hours, 30°C).

References

Karavaeva, Iuliia E., Sergey A. Golyshev, Ekaterina A. Smirnova, Svyatoslav S. Sokolov, Fedor F. Severin, and Dmitry A. Knorre. 2017. “Mitochondrial Depolarization in Yeast Zygotes Inhibits Clonal Expansion of Selfish mtDNA.” *Journal of Cell Science* 130 (7): 1274–84.
